# Association between isotretinoin use and central retinal vein occlusion in an adolescent with minor predisposition for thrombotic incidents: a case report

**DOI:** 10.1186/1752-1947-3-58

**Published:** 2009-02-10

**Authors:** Georgios Labiris, Andreas Katsanos, Maria Karapetsa, Ioanna Mpanaka, Dimitrios Chatzoulis

**Affiliations:** 1 Ophthalmology Department, University Hospital of Larissa, 41110 Larissa, Greece; 2 Internal Medicine Department, University Hospital of Larissa, 41110 Larissa, Greece

## Abstract

**Introduction:**

We report an adolescent boy with minimal pre-existing risk for thromboses who suffered central retinal vein occlusion associated with isotretinoin use for acne. To the best of our knowledge, this is the first well documented case of this association.

**Case presentation:**

An otherwise healthy 17-year-old white man who was treated with systemic isotretinoin for recalcitrant acne was referred with central retinal vein occlusion in one eye. Although a detailed investigation was negative, DNA testing revealed that the patient was a heterozygous carrier of the G20210A mutation of the prothrombin gene. Despite the fact that this particular mutation is thought to represent only a minor risk factor for thromboses, it is probable that isotretinoin treatment greatly increased the risk of a vaso-occlusive incident in this patient.

**Conclusion:**

Isotretinoin use may be associated with sight- and life-threatening thrombotic adverse effects even in young patients with otherwise minimal thrombophilic risk. Physicians should be aware of such potential dangers.

## Introduction

Isotretinoin, a vitamin A derivative, is a synthetic retinoid used for the treatment of severe cystic acne that does not respond to other therapies. The drug appears to act by inhibiting sebaceous gland size and function. Besides being teratogenic, a number of adverse effects have been described for isotretinoin [1-3]. The most common ones include dryness and itching of the skin and mucous membranes. Less commonly reported adverse effects are headache, inflammatory bowel disease, anorexia, alopecia, pseudotumour cerebri, muscle and joint pains, as well as premature closure of epiphyseal growth plates in children's joints. An increase in serum lipid levels is also frequently seen [4]. Previous reports have indicated an association between isotretinoin use and thrombotic, thromboembolic or haemorrhagic events whereas the Canadian Adverse Reaction Newsletter described 11 such cases of thromboembolic incidents, strokes and myocardial infarctions for the period 1983–2005 [1-3].

## Case presentation

A 17-year-old white man was referred by his ophthalmologist to the University Department of Ophthalmology in Larissa, Greece, with the diagnosis of central retinal vein occlusion (CRVO) in his left eye. The patient's ophthalmic history was negative, whereas his general medical history was only significant for acne, for which he had been treated with oral isotretinoin 20 mg three times daily (13-*cis*-retinoic acid, *Accutane*^®^) for the previous 6 weeks. He denied smoking, alcohol consumption and illicit substance use. The patient successfully participated in all regular sports activities at school, and presented a normal body mass index of 23.77. Besides cheilitis with dry, cracked and crusted lips, his initial physical examination was negative.

**Figure 1:**
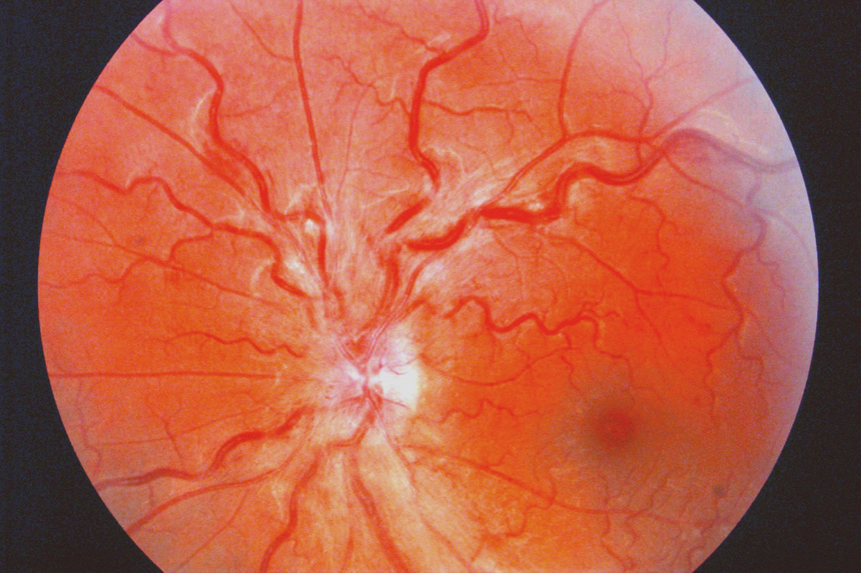
**Funduscopic image of the left eye**. Fundus photograph of the patient's left eye with optic disc oedema, retinal haemorrhages and engorged, tortuous veins. Photograph taken on the day of presentation.

**Figure 2:**
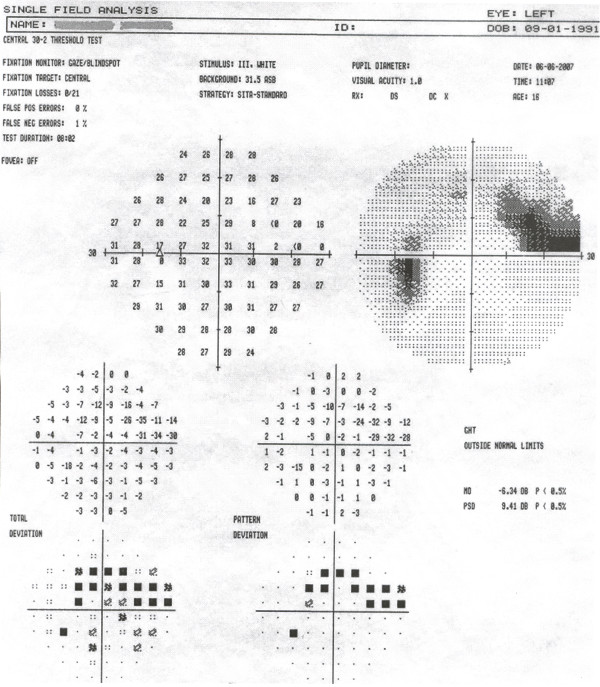
**Visual field of the left eye**. Printout of the visual field test of the patient's left eye exhibiting a superior arcuate scotoma. Test performed 2 days after presentation.

His uncorrected visual acuity was 12/10 in each eye and the intraocular pressure in his right and left eye was 14 and 17 mmHg, respectively. Funduscopy revealed optic disc oedema with retinal haemorrhages and engorged, tortuous veins in the left eye (Figure 1). His right eye had a normal fundus with an optic nerve head having a cup-to-disc ratio of 0.4. Visual fields examination revealed a superior arcuate scotoma in his left eye (Figure 2).

A detailed clinical investigation of all systems, including cardiovascular and neurological assessment was unremarkable. Total blood count with differential, erythrocyte sedimentation rate (ESR), C-reactive protein (CRP), and routine laboratory testing were within normal ranges, except for a mild increase in low-density lipoprotein (LDL) cholesterol that was attributed to isotretinoin use. Urine analysis and 24-hour urine selection specimens were within normal ranges. Moreover, no viral or other systemic or localised infection was detected. Further investigation with plasma protein electrophoresis, autoimmune and tumour markers, screening for antiphospholipid syndrome, and cryoglobulinaemia was also negative. Finally, the patient presented normal values of protein C, protein S, antithrombin, and homocysteine. DNA testing for potential genetic thrombophilic predisposition revealed that the patient was a heterozygous carrier of the G20210A mutation of the prothrombin gene (Table 1). However, his family history was negative for thrombotic incidents even for senior relatives (grandparents). On the other hand, chest computed tomography (CT), abdominal ultrasound, brain and orbit magnetic resonance imaging (MRI) scans, as well as brain and orbit magnetic resonance angiography (MRA), were all negative.

**Table 1:** DNA testing for genetic predisposition to hypercoagulability states

Mutation tested	Result
FII G20210A	Heterozygous G->A
FV G1691A	Normal
MTHFR C677T	Heterozygous C->T
MTHFR A1298C	Normal
PAI-1 (-675 & -844)	Normal

Isotretinoin treatment was discontinued and the patient was initially given low molecular weight heparin, followed by oral anticoagulants (acenocoumarol, *Sintrom*^®^). Based on the notion that elevated intraocular pressure may be a risk factor for CRVO, intraocular pressure lowering medication was administered (brimonidine BID, *Alphagan*^®^). Due to the patient's slow response to the treatment, systemic steroids were added to the therapeutic scheme (methylprednisolone sodium acetate 500 mg intravenously for 3 days, then oral methylprednisolone 24 mg once daily for 1 month with gradual dosage decrease). Six months after the CRVO, the optic disc oedema had regressed and the haemorrhages had been absorbed. The patient's visual acuity remains 12/10 without signs of posterior- or anterior segment neovascularisation, whereas the visual field defect has slightly decreased in depth. Cheilitis was attributed to isotretinoin and resolved gradually after its discontinuation.

## Discussion

Regarding the ocular adverse effects related to isotretinoin [5,6], they can be categorised into the following classes according to the World Health Organization classification for causality of suspected drug-related events: "certain", "probable/likely", "possible", "unlikely", "conditional/unclassified" and "inaccessible/unclassifiable". Thus, the "certain" category includes abnormal meibomian gland secretion and atrophy, intracranial hypertension with optic disc oedema, ocular sicca, corneal opacities, keratitis, myopia and decreased dark adaptation. The "probable/likely" category includes reversible decreased colour vision and permanent loss of dark adaptation. Adverse events that have a "possible" association with isotretinoin are permanent sicca, corneal ulcers, diplopia and eyelid oedema. The "unlikely" category is comprised of entities such as exophthalmos, keratoconus, glaucoma, activation of herpes simplex and pupil abnormalities. The "conditional/unclassified" and "inaccessible/unclassifiable" categories include a variety of events for which data are insufficient or contradictory. It is noteworthy that isotretinoin can have a significant effect on the cornea and the ocular tear film [7,8]; this is of particular clinical relevance because the age distribution of patients treated with isotretinoin overlaps to a large extent with the age distribution of patients undergoing very popular corneal refractive operations.

Regarding the patient presented in this report, although the MTHFR C677T mutation is not associated with a thrombotic diathesis, heterozygosity in the G20210A mutation is considered to be a minor predisposing factor for thrombotic incidents in otherwise healthy young adults. However, the introduction of isotretinoin treatment possibly initiated or facilitated the thrombotic process. Besides previous reports that indicated an association between isotretinoin use and thrombotic, thromboembolic or haemorrhagic events [1,2], the Canadian Adverse Reaction Newsletter described 11 such cases of thromboembolic incidents, strokes and myocardial infarctions for the period 1983–2005 [3]. Nine of the patients were aged 29 or younger, whereas four of the 11 patients had no other risk factor. Paradoxically, some reports indicate a possible protective effect of isotretinoin in thromboembolic disorders. Some of the underlying mechanisms may be the decrease in lipoprotein (a) which has been implicated in coronary heart disease and stroke and the inhibition of vascular smooth muscle proliferation and vessel remodelling [9,10]. Thus, the drug appears to act on the coagulation process by a still unexplained mechanism.

Considering our patient, the relationship between isotretinoin intake and CRVO is "probable" both according to the Naranjo probability scale [11] and the World Health Organization classification for causality of drug-related reactions.

## Conclusion

Oral isotretinoin treatment was associated with central retinal vein occlusion in our adolescent male patient who only had a minor genetic predisposition for thrombosis. Although the occurrence of this sight-threatening adverse event is rare, there is a probable relationship between isotretinoin intake and CRVO. The risk of thrombotic incidents even in young patients should be kept in mind by prescribing physicians.

## Abbreviations

CRP: C-reactive protein; CRVO: central retinal vein occlusion; CT: computed tomography; DNA: deoxyribonucleic acid; ESR: erythrocyte sedimentation rate; LDL: low-density lipoprotein; MRA: magnetic resonance angiography; MRI: magnetic resonance imaging

## Consent

Written informed consent was obtained from the patient for publication of this case report and any accompanying images. A copy of the written consent is available for review by the Editor-in-Chief of this journal.

## Competing interests

The authors declare that they have no competing interests.

## Authors' contributions

GL was involved in the ophthalmic management of the patient and contributed to writing the manuscript. AK performed some of the ophthalmic examinations and was involved in writing and reviewing the manuscript. MK carried out part of the general medical work-up and the genetics investigation. IM performed the general clinical investigation. DC was involved in the ophthalmic evaluation of the patient and critically reviewed the paper. All authors read and approved the final manuscript.
